# Comparative analysis of cervical cancer classification of DPAGCHE-enhanced Pap smear images using convolutional neural network models

**DOI:** 10.1371/journal.pone.0330103

**Published:** 2025-09-08

**Authors:** Khalis Khiruddin, Wan Azani Mustafa, MD Ashequl Islam, Khairur Rijal Jamaludin, Hiam Alquran, Khairul Shakir Ab Rahman

**Affiliations:** 1 Faculty of Electrical Engineering & Technology, Universiti Malaysia Perlis, Pauh Putra Campus, Arau, Perlis, Malaysia; 2 Advanced Computing (AdvComp), Centre of Excellence (CoE), Universiti Malaysia Perlis (UniMAP), Campus Pauh Putra, Arau, Perlis, Malaysia; 3 Faculty of Mechanical Engineering & Technology, Universiti Malaysia Perlis, Kampus Tetap Pauh Putra, Arau, Perlis, Malaysia; 4 Faculty of Artificial Intelligence, Universiti Teknologi Malaysia, Jalan Sultan Yahya Petra, Kuala Lumpur, Malaysia; 5 Department of Biomedical Systems and Informatics Engineering, Yarmouk University, Irbid, Jordan; 6 Department of Pathology, Hospital Tuanku Fauziah, Jalan Tun Abdul Razak, Kangar, Perlis, Malaysia; SRM-RI: SRM Institute of Science and Technology (Deemed to be University) Research Kattankulathur, INDIA

## Abstract

Cervical cancer remains a significant cause of female mortality worldwide, primarily due to abnormal cell growth in the cervix. This study proposes an automated classification method to enhance detection accuracy and efficiency, addressing contrast and noise issues in traditional diagnostic approaches. The impact of image enhancement on classification performance is evaluated by comparing transfer learning-based Convolutional Neural Network (CNN) models trained on both original and enhanced images. This study employs transfer learning with pre-trained CNNs to classify preprocessed Pap smear images into three categories. Data augmentation, including rotations, flips, and shifts, enhances variability and prevents overfitting. The OneCycle learning rate schedule dynamically adjusts the learning rate, improving training efficiency. To enhance image quality, the Denoised Pairing Adaptive Gamma with Clipping Histogram Equalization (DPAGCHE) method improves contrast and reduces noise. The evaluation involves five pre-trained CNN models and the publicly available Herlev dataset, implemented in MATLAB Online. The ResNet50 model trained on the DPAGCHE-enhanced dataset achieves the highest classification performance, with 84.15% accuracy, along with improved specificity, recall, precision, and F1-score. ResNet50’s residual connections mitigate vanishing gradient issues and enhance deep feature extraction. Accordingly, the DPAGCHE preprocessing significantly improves classification performance, leading to a 53.65% increase in F1-score and 44.29% in precision. In contrast, the Baseline CNN reaches only 66.67% accuracy, highlighting the advantage of deeper architectures combined with enhanced preprocessing. These findings suggest integrating DPAGCHE-enhanced preprocessing with deep learning improves automated cervical cancer detection. In particular, ResNet50 demonstrates the best performance, reinforcing the effectiveness of contrast enhancement and noise reduction in aiding classification models.

## Introduction

In the cells of the cervix, cervical cancer originates from the lower portion of the uterus that is anatomically connected to the vagina. It occurs when the cervix cells increase abnormally, as stated by Sausen et al. [1] and Guimarães et al. [2]. An estimated 660,000 new cases of cervical cancer and 350,000 deaths occurred globally in 2022, according to the World Health Organization [3]. Notably, cervical cancer is a common form of the disease that likely every woman will eventually develop. Furthermore, it is associated with a high mortality rate, as stated by Mustafa et al*.* [4,5]. Based on the paper by the same author [6], the Papanicolaou test developed during the 1940s is also known as the Pap test or Pap smear, which enables microscopic analysis of these cells to detect pre-cancerous or cancerous caries. The traditional method of cervical cancer screening is based on the Pap smear test. However, Macios et al. and Schiffman et al. stated in their studies [7] and [8] that this method has several limitations. This includes low recall and specificity, which can lead to false-negative or false-positive results.

Cell classification has become an essential step in diagnosing and understanding the progression of cervical cancer. Mustafa et al. noted in their paper [4] that the adoption of Computer-Aided Detection (CAD) technologies has revolutionized cervical cancer screening. Despite technological advancements significantly enhancing early detection, Alias et al. emphasized in their paper [9] that accurate diagnosis remains challenging for various reasons. Note that classifying cervical cells in cytology Pap smear images is critical for automated cervical cancer screening. However, the relatively low accuracy of these complex techniques for abnormal cell classification hampers their effectiveness in automatic diagnosis. The rise of artificial intelligence, particularly deep learning approaches, has demonstrated great promise in computer-aided diagnosis. These methods can automatically extract visual features with high precision and low error [10], significantly improving classification accuracy. Remarkably, this advancement has been widely applied in numerous studies for cell classification.

This study aims to contribute to a more effective approach to identifying abnormal cells through the use of deep learning models. By leveraging pre-trained Convolutional Neural Networks (CNNs) and employing advanced data augmentation techniques, we seek to enhance the accuracy and reliability of automated cervical cancer detection. The main contributions of the present study are as follows:

a. This research simplifies the classification categories of cervical cells compared to most studies, with the aim of increasing precision while maintaining the clinical relevance of the results. By classifying cervical cells according to medical grade categories, the study enhances the accuracy, precision, and F1-score without sacrificing the applicability of automated cervical cancer detection systems.
b. This study incorporates the OneCycle Learning Rate strategy into the algorithm, optimizing the training process. The OneCycle Learning Rate adjusts the learning rate dynamically, leading to faster convergence and improved model performance, as we discovered no previous research applying the OneCycle Learning Rate to cervical cancer classification.
c. Unlike previous studies, this research minimizes the preprocessing steps by applying a low-light contrast enhancement with a dynamic contrast adjustment novel method adapted. Although most studies incorporate feature extraction and cell segmentation, our approach focuses on enhancing image quality directly to improve the performance of the classification model.

## Related works

The advent of deep learning, CNNs, has enabled automated cervical cell classification, offering faster and more accurate solutions [11,12]. Correspondingly, this paper reviews several deep learning-based research studies conducted over the past five years, focusing on their methodologies, performance, and challenges.

### Hybrid deep feature fusion approaches

Hybrid deep-learning models have gained popularity due to their ability to improve feature extraction and classification accuracy. Rahaman et al. [11] introduced Deep Cervix, a hybrid model integrating CNN-based feature extraction for binary and multiclass cervical cell classification. The model achieved accuracies of 99.85% and 99.14% for binary and five-class classification on the SIPaKMeD dataset, respectively. Despite these high accuracies, challenges related to imbalanced datasets persisted.

Similarly, Li et al. [13] proposed a deep fusion approach to combine local and global features, enhancing classification performance. This approach demonstrated increased recall to cervical abnormalities but at the cost of higher computational complexity. Meanwhile, Aurna et al. [14] addressed these limitations by employing a two-stage hierarchical CNN for multiclass classification. Their model improved classification accuracy yet struggled with robustness when applied to noisy datasets. At the same time, Kim et al. [15] tackled this issue by incorporating noise-reduction techniques into a hybrid deep-learning framework, resulting in enhanced noise tolerance.

### Multi-architecture integration

The integration of multiple deep learning architectures has proven to enhance feature extraction and improve classification accuracy. Liu et al. [16] proposed the CVM-Cervix framework, which combines CNNs, Vision Transformers (ViTs), and Multilayer Perceptrons (MLPs) to capture complementary feature sets. This model achieved high classification accuracy, although its increased training time and computational resource requirements posed challenges. Furthermore, Chen et al. [17] addressed class imbalances by integrating CNN with Generative Adversarial Networks (GANs), achieving improved classification accuracy on imbalanced datasets. However, instability during GAN training remained a concern.

Other studies, including Chen et al. [18], demonstrated the effectiveness of combining DenseNet and InceptionV3 models for improved feature extraction. Although these hybrid approaches enhanced recall and specificity, they also increased computational demands and reduced interpretability.

### Semi-supervised and attention-based models

To address the challenge of limited labeled cervical cell data, several studies employed semi-supervised learning. Manivannan et al. [19] introduced a semi-supervised approach that leverages both labeled and unlabeled data, improving classification accuracy while reducing manual annotation requirements. Despite that, managing training complexities due to the inclusion of unlabeled data posed challenges.

Attention-based models have also gained traction for their ability to enhance interpretability. Building on this, Wang et al. [20] developed an interpretable attention network that highlights critical regions in cervical cell images, improving both classification accuracy and model explainability. Al-Masni et al. [21] further refined this approach by implementing dual attention mechanisms to improve robustness to image noise, although the increased computational overhead remained an issue.

### CNN-based classification and simplified architectures

CNNs remain central to cervical cell classification research due to their proven ability to learn spatial features from image data. Jafari et al. [22] developed DeepPap, a CNN-based model that classified cervical cells without explicit segmentation. This approach addressed challenges related to overlapping cells but required large training datasets to avoid overfitting.

Alem et al. [23] used hyperparameters and early stopping techniques to apply an end-to-end CNN Feature Extractor (CNN-FE) model for Land Cover/Land Use (LCLU) classification in the UC-Merced dataset. This approach reduced computational complexity, although it required larger datasets for optimal performance. Simultaneously, Verma et al. [24] proposed a lightweight CNN for real-time classification, demonstrating reduced computational demands while maintaining high accuracy.

Related studies by Wubineh et al. [25] and Gan et al. [26] highlighted the performance of ResNet50 and DenseNet in cervical cell classification tasks. Their findings emphasized the significance of pre-trained models and transfer learning for improving generalization on smaller datasets.

## Methods

**Fig 1:**
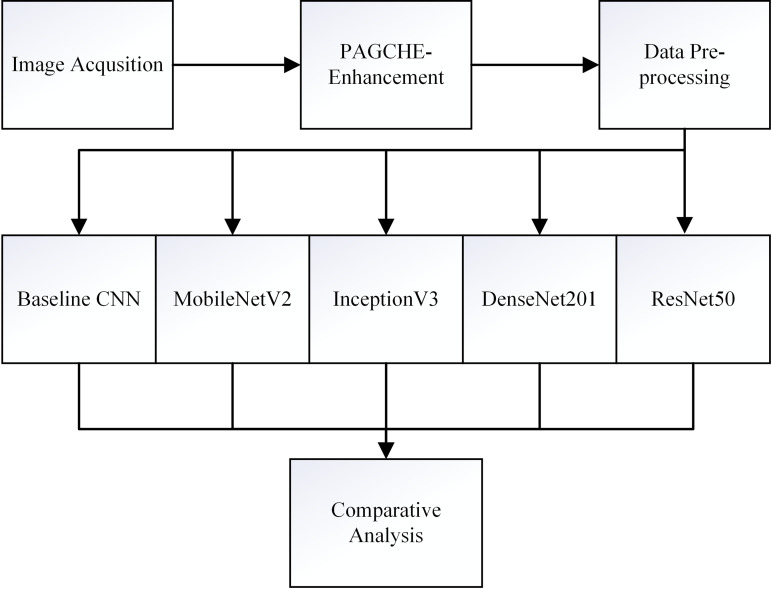
Workflow for the multiclass cervical cell classification framework. Workflow for the proposed multiclass cervical cell classification framework. The process includes image acquisition, DPAGCHE-based preprocessing for contrast enhancement and denoising, followed by classification using transfer learning with five CNN models, and concludes with comparative performance analysis. This pipeline aims to automate and enhance cervical cancer screening by improving feature quality and model accuracy.

This study evaluated and compared five CNN models on the public Herlev dataset for three-class classification of cervical cancer cells. All processes, including data acquisition, enhancement, preprocessing, classification, and performance analysis, were conducted using MATLAB Online, facilitating the seamless implementation of the proposed deep learning pipeline. Fig 1 illustrates the multiclass cervical cell classification framework, highlighting key stages such as image preprocessing, model training, and evaluation. This framework aims to enhance the accuracy and reliability of cervical cell image classification by leveraging deep learning architectures and comparing their performance using multiple evaluation metrics.

The process begins with Data Acquisition, where cervical cell images are gathered from relevant datasets. Subsequently, Denoised Pairing Adaptive Gamma with Clipping Histogram Equalization (DPAGCHE), a specialized preprocessing technique, is applied to improve image quality by enhancing clarity and contrast as well as reducing noise. The enhanced images are then subjected to Data Preprocessing, which includes resizing and data splitting. Once pre-processed, the images are passed through five different deep learning models: Baseline CNN, MobileNetV2, InceptionV3, DenseNet201, and ResNet50. Each model was selected based on its distinct architectural properties and capability to extract hierarchical and complex image features critical for multiclass classification.

In the final stage, the outputs of these CNN models undergo Comparative Analysis, where their classification performance is evaluated using key metrics: accuracy, specificity, recall, precision, and F1-score. These metrics provide comprehensive insights into each model’s effectiveness in distinguishing normal cervical cells from abnormal ones (including High-Grade Squamous Intraepithelial Lesion (HSIL) and Low-Grade Squamous Intraepithelial Lesion (LSIL) categories) and minimizing false positives and false negatives.

### Image acquisition

Notably, 917 sample images were collected from the Herlev database (Herlev University Hospital, Denmark). The database was obtained from/developed by NiSIS (EU coordination action, contract 13569), a Nature-inspired Smart Information System, with particular significance for the Nature-Inspired Data Technology focus group. It is thus accessible (http://fuzzy.iau.dtu.dk/download/smear2005) on the World Wide Web.

**Fig 2:**
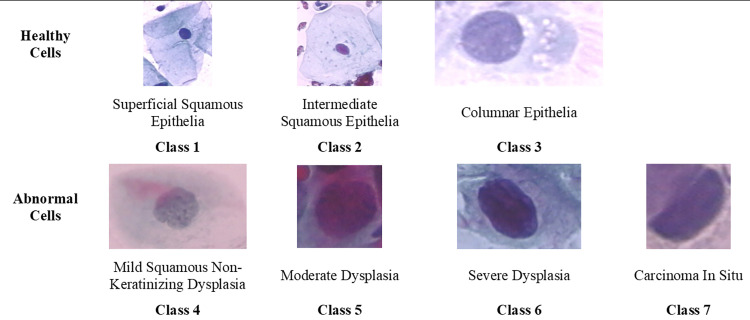
Original classification of herlev dataset. Original classification of the Herlev dataset, consisting of 7 classes: 3 healthy (superficial, intermediate, columnar) and 4 abnormal (mild, moderate, severe dysplasia, carcinoma in situ), used for cervical cell image analysis.

The Herlev dataset provides images of cervical cells, categorized into three classes of normal cells (242 images) and four classes of abnormal cells (675 images). In particular, the normal cells include superficial squamous (74 images), intermediate squamous (70 images), and columnar cells (98 images). Conversely, the abnormal cells are categorized into mild dysplasia (193 images), moderate dysplasia (146 images), severe dysplasia (197 images), and carcinoma in situ (150 images). However, it is essential to note that the images are unevenly distributed across these classes. Additionally, the ratio and size of the images vary significantly, as displayed in Fig 2.

**Fig 3:**
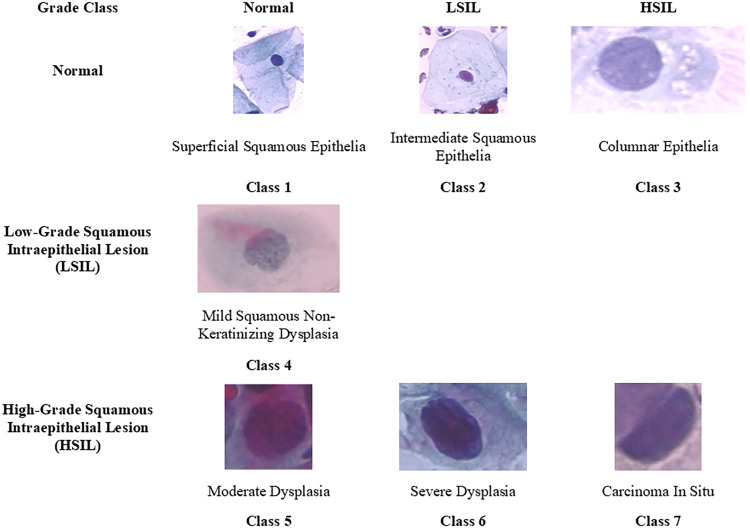
Herlev database distribution according to SIL classification. Redistribution of the original 7 Herlev classes into 3 categories—Normal, LSIL, and HSIL—based on the Bethesda System (SIL classification).

To streamline the analysis, these categories have been manually redistributed using the Squamous Intraepithelial Lesion (SIL) classification. This redistribution is depicted in Fig 3, which displays how the seven original classes are grouped into three classes: Normal, LSIL, and HSIL. Specifically:

- Normal Class: Consists of superficial squamous, intermediate squamous, and columnar cells.
- LSIL Class: Includes mild dysplasia.
- HSIL Class: Comprises moderate dysplasia, severe dysplasia, and carcinoma in situ.

Redistribution also aids in enhancing classification performance while preserving the clinical relevance of the results since the SIL classification is utilized in clinical practice. Thus, by aligning the dataset with clinically relevant classifications, the model can provide more accurate and actionable insights that are directly applicable in a medical context.

### Image contrast enhancement

This study proposed two steps: denoise and color contrast enhancement. The input images were converted to the color space of the Hue, Saturation, Value (HSV) system. The HSV color space is widely used for color and illumination analysis as it closely aligns with human color perception [27]. In this study, the V (value) channel is extracted and processed with a median filter (15 × 15 window size) to remove salt-and-pepper noise. However, applying a median filter can lead to contrast degradation. To address this, the Pairing Adaptive Gamma Correction and Histogram Equalization (PAGCHE) method, originally proposed by Bataineh [27], is integrated to enhance pixel contrast.

**Fig 4:**
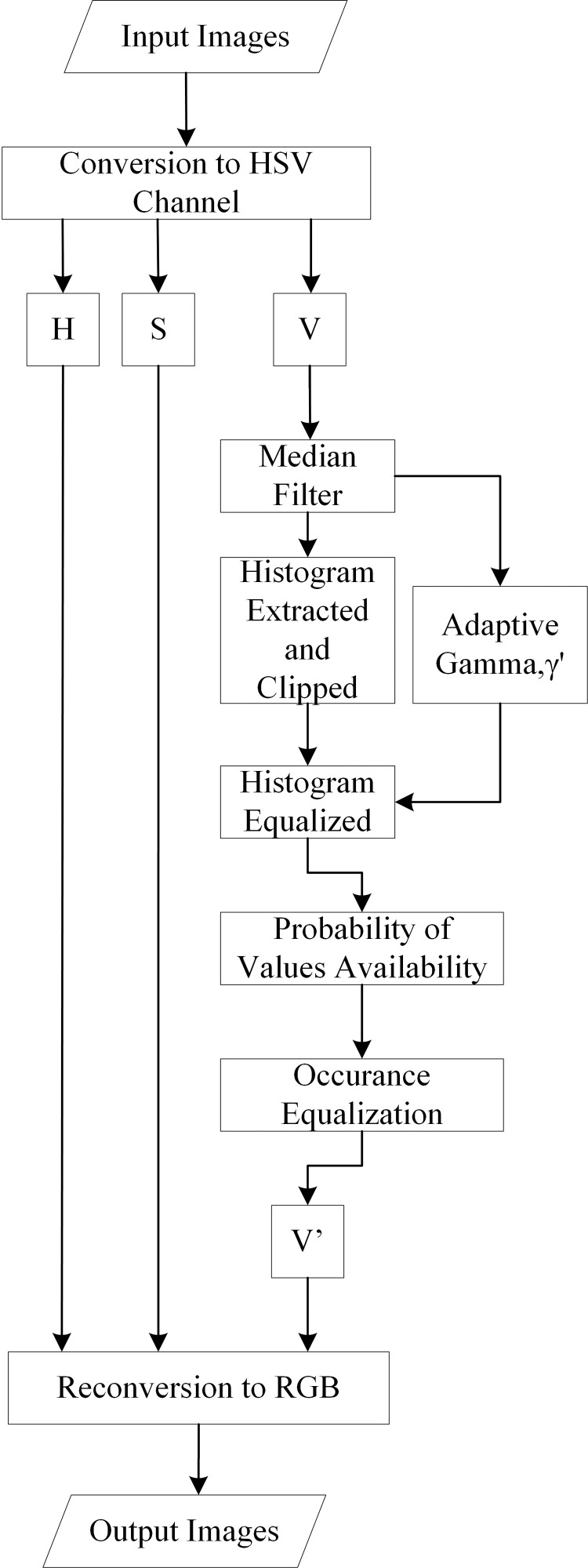
Flowchart of image contrast enhancement.


In this study, the median filter is incorporated as a preprocessing step to improve the denoising capability of PAGCHE. This modified approach is referred to as DPAGCHE, ensuring both noise suppression and contrast enhancement for improved image quality. Referring to Fig 4, gamma and histogram will be extracted from the V channel of the input images. An adaptive gamma generator calculates gamma parameters (γ’) based on the image’s dark, medium, or bright conditions using the following proposed equation in Equation 1 below:

$$\gamma^{\prime }=\frac{\text{Mean}}{\text{L}\times \text{0}.\text{33}}+\text{C,}$$ (1)

where L is the maximum grey level of the images. Here, L = 255, and it is multiplied by 0.33 to adapt the $\gamma^{\prime }$ value to one of the three global states, which are dark, medium, or bright, for the images. C is a bias constant to overcome the problem of $\gamma^{\prime }$; here, C = 0.1. The computed gamma parameters propose a Cumulative Distribution Function (CDF) that optimizes the illumination values using Equation 2 below:


$$\begin{array}{c} cdf^{\prime }(l)={\left(\sum {\mathrm{\backslash nolimits}}_{l=0}^{l=L}pdf(l)\right)}^{\gamma \prime }, \end{array}$$ (2)

where $cdf^{\prime }$ is optimized CDF, pdf is the probability distributive function, and l is the intensity value. Next, a significant modification of Histogram Equalization (HE) is performed to improve image contrast and prepare the illumination for subsequent stages. At the same time, a proposed CDF function is used to greatly enhance the illumination. This function aims to improve the illumination and contrast adaptively to unify the visual properties of any image, ranging from considerably light to dark. New values of the brightness matrix are calculated using the processed CDF value as the following Equation 3 below:


$$V_{MID}(x,y)=cdf\prime (V(x,y))\times 255$$ (3)

After calculating the $V_{MID}$ matrix, a second equalization process is applied to modify the displacement of values evenly. Instead of using a standard pdf, an equal density probability is used here. The distribution of displaced values in the $V_{MID}$ matrix is corrected by rearranging equally to correct the illumination levels and contrast of the processed images. The result is a processed brightness matrix with improved contrast and illumination properties for the original V channel.

### Data preprocessing

The Herlev database was loaded and formatted to match the input dimensions required by the CNN models. To mitigate the risk of overfitting and ensure effective training, the dataset was partitioned into three subsets: training set (80%), validation set (20%), and testing set (10%). The testing set was exclusively used to evaluate the models after completing the training phase. In addition, data augmentation techniques were applied to both the training and validation datasets to increase variability and improve model generalization. This augmentation involved applying transformations such as random rotations, horizontal and vertical flips, zooming, and shifting. Accordingly, these transformations artificially expand the dataset, allowing the models to better oversee variations in cervical cell images and enhancing classification robustness.

### Classification

A CNN is a specialized type of neural network widely utilized in the field of image recognition [28,29]. The basic structure of a CNN typically comprises an input layer, two convolutional layers, two pooling layers, two fully connected layers, and an output layer, making up a total of eight layers [30]. Let the *m-th* input feature map to the convolutional layer be denoted as $X_{m}$, and $W_{n,m}$ represent the convolution kernel connecting the *m-th* input feature map to the *n*-th feature map in the current layer. The output $y_{n}$ of the *n-th* feature map in the convolutional layer can then be expressed as:


$$y_{n}=f\left(\sum_{m}X_{m}*W_{n,m}+b_{n}\right).$$ (4)

Here, $b_{n}$ represents the bias parameter of the *n*-th feature map in the current layer, while * denotes the discrete convolution operation. The function f is the activation function, commonly a non-linear mapping [31].

Pooling operations, which include max pooling and average pooling, are essential components of CNNs, helping reduce the spatial dimensions of feature maps to mitigate overfitting and enhance optimization efficiency [32]. After convolution operations extract relevant features, these features can be used to train the classifier. One common classifier is the Softmax classifier, though it can face challenges related to computational complexity. For instance, when processing a 96 × 96 pixel image and learning 400 features defined on an 8 × 8 input, each feature-map-to-image convolution results in a (96–8 + 1) × (96–8 + 1) = 7921-dimensional convolution feature. Consequently, with 400 features, each image example produces a 7921 × 400 = 3,168,400-dimensional convolution feature vector.

If the image input to the pooling layer is denoted as $x^{\left(l-1\right)}$, and the image output after pooling is $x^{\left(l\right)}$ and down() is the downsampling operation, which typically reduces the spatial dimensions of the feature map by applying operations such as max pooling or average pooling, the pooling operation can be represented mathematically as:


$$x^{\left(l\right)}=down\left(x^{\left(l-1\right)}\right)$$ (5)

The fully connected layer is at the tail of the CNN. It converts the two-dimensional feature map of the convolution output into a one-dimensional vector, that is, connects all the features and finally sends the output value to the classifier, such as the Softmax classifier [33]. After Softmax, the output can be expressed as:


$${S(y)}_{i}=\frac{e^{yi}}{\sum_{j=1}^{n}e^{yi}},$$ (6)

where ${S(y)}_{i}$ represents the probability of class iii in the final classification output. yi is the raw score (logit) for class iii before applying Softmax. Meanwhile, $e^{yi}$ is the exponentiation of the logit value, ensuring all outputs are positive. Moreover, $\sum_{j=1}^{n}e^{yi}$ is the sum of exponentiated logits for all n classes, serving as a normalization factor to ensure the output probabilities sum to 1.

This study employed five prominent CNN architectures, ResNet50, DenseNet201, InceptionV3, MobileNetV2, and a Baseline CNN, each with distinct architectural features and strengths suited for image classification tasks. These models were selected for their proven capabilities in handling complex feature extraction from raw Pap smear images, minimizing the need for manual segmentation or handcrafted feature engineering. Notably, by leveraging the end-to-end learning capability of these deep learning models, this study aimed to improve cervical cell classification accuracy.

To provide a clearer comparison of these CNN architectures and their performance trade-offs, Table 1 presents an overview of key characteristics, including the number of layers, parameters, notable architectural features, strengths, and limitations. This table highlights the diversity in design strategies employed by each model and their potential impact on classification performance.

**Table 1:** Architectural comparison of CNN models for cervical cell classification. Architectural comparison of five CNN models used in this study, highlighting their parameter size, key design features, strengths, and limitations. The table outlines the trade-offs between computational complexity and classification capability, informing the selection of models suited for Pap smear image classification.

Model	Number of parameters	Key architectural features	Strengths	Limitations
**ResNet50**	~23.5 million	Residual connections with skip layers to prevent vanishing gradients	Effective for deep networks, hierarchical feature extraction	High memory and computational requirements
**DenseNet201**	~20.2 million	Densely connected layers (each layer connected to every preceding layer)	High feature reuse, reduced number of parameters	Increased computational complexity
**InceptionV3**	~22 million	Inception modules (parallel convolutions with 1x1, 3x3, and 5x5 filters)	Multi-scale feature learning, computational efficiency	Requires careful architectural design
**MobileNetV2**	~3.4 million	Depthwise separable convolutions, inverted residuals with linear bottlenecks	Lightweight, optimized for mobile/embedded devices	Slightly lower accuracy on complex datasets
**Baseline CNN**	1-2 million	Two convolutional layers, two pooling layers, fully connected layers	Simple, faster training, suitable for small datasets	Limited feature extraction capability, prone to overfitting

ResNet50 is a deep CNN architecture comprising 50 layers, distinguished by its use of residual connections. These connections bypass certain layers, effectively addressing the vanishing gradient problem that often arises in deep networks [34]. Furthermore, by enabling uninterrupted gradient flow, ResNet50 allows for efficient training of deep architectures. It also excels in extracting hierarchical features from complex medical images, making it well-suited for tasks like cervical cancer classification [35].

DenseNet201, on the other hand, employs densely connected architecture featuring dense blocks where every layer is connected to every other layer. This design promotes feature reuse and ensures efficient gradient flow throughout the network. In addition, DenseNet201 is computationally efficient and excels at extracting rich feature representations with fewer parameters [36,37]. In the study, DenseNet201 emerged as the best-performing model, achieving the highest classification accuracy due to its ability to capture intricate patterns in Pap smear images.

InceptionV3 is designed for computational efficiency and multi-scale feature learning. Its unique inception modules process input features at multiple scales simultaneously using parallel convolutional layers of different sizes [38]. This approach enables InceptionV3 to balance computational complexity and classification accuracy [39]. This study demonstrated strong performance across several classes, particularly in capturing multi-scale patterns within the cervical cell images.

MobileNetV2 is a lightweight CNN model optimized for mobile and edge devices. It uses depthwise separable convolutions and inverted residual bottleneck layers to significantly reduce computational requirements while maintaining accuracy [40]. MobileNetV2 is particularly advantageous in scenarios with constrained resources, offering fast training times and efficient inference [41]. Although it is less complex than DenseNet201 and InceptionV3, it presented strong results, making it a practical choice for real-world deployment.

The Baseline CNN, a custom-built network with a straightforward structure, serves as a benchmark in this study. It includes basic convolutional layers, pooling layers, and fully connected layers, providing a simpler alternative to the more advanced pre-trained architectures. While the Baseline CNN is less sophisticated, it establishes foundational performance metrics, highlighting the comparative strengths of the more complex models [42,43].

**Algorithm 1. Pseudocode of training and evaluation of transfer learning.**

 Procedure

  Step 1: Load and Prepare Data

   LOAD images from dataDir

   SPLIT images into trainImds and valImds

  Step 2: Data Augmentation

   AUGMENT trainImds

   AUGMENT valImds

  Step 3: Define the Model

   FOR each model IN [ResNet50, DenseNet201, InceptionV3, MobileNetV2, Baseline CNN]

   LOAD pre-trained model

   MODIFY model for transfer learning with new fully connected layer and classification layer

   CREATE layer Graph from the model

  Step 4: Set Up Training Options

   SET training options with OneCycle learning rate schedule

   % Start timing

   START timer

   % Train the model

   TRAIN model with augmented TrainImds and training options

   STOP timer

   PLOT training and validation accuracy and loss

  Step 5: Evaluate the Model

   CLASSIFY images in augmentedValImds

   CALCULATE confusion matrix

   CALCULATE Accuracy specificity, recall, precision, F1-score

   % Display metrics

   PRINT accuracy, specificity, recall, precision, F1-score

   % Save metrics to Excel

   SAVE metrics to ‘results.xlsx’

   SAVE confusion matrix to ‘results.xlsx’

  END FOR

 END

Although several state-of-the-art models exist, including EfficientNet, VGG, and Vision Transformer (ViT)-based architectures, they were not included in this study for the following reasons. First, EfficientNet and transformer-based models typically require larger datasets and significant computational resources for optimal performance, which may not be feasible within the constraints of the current study using the relatively small Herlev dataset. Second, VGG models, while historically influential, are computationally less efficient and often outperformed by more recent architectures such as ResNet and DenseNet in both accuracy and training efficiency. The selection of ResNet50, DenseNet201, InceptionV3, and MobileNetV2 represents a balance between model diversity, computational efficiency, and proven performance in similar medical image classification tasks. Nonetheless, future work could include these excluded models for broader benchmarking or hybrid integration to explore potential improvements in accuracy and generalizability.

## Results and discussion

This section analyzes the evaluation metrics for two datasets (original and DPAGCHE-enhanced) processed using five different CNN models. Next, the imbalance class problem on classification tasks is validated. Lastly, a comparison study is conducted to study the differences in the performance of all the studied models.

### Result of DPAGCHE-enhanced on dataset

**Fig 5:**
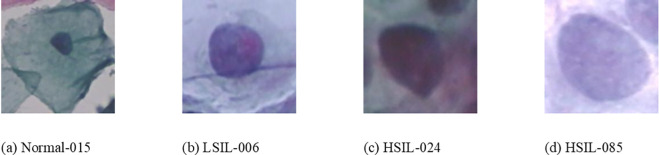
Random selection of original image dataset. Randomly selected images from the original Herlev dataset, representing three classes: Normal-015, LSIL-006, HSIL-024, and HSIL- 085. These samples exhibit low contrast, noise presence, and poor nucleus-cytoplasm differentiation, which may hinder accurate feature extraction by deep learning models. This visual limitation highlights the need for image enhancement prior to classification.

**Fig 6:**
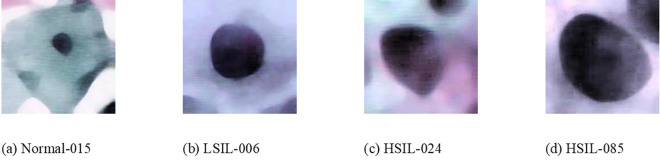
Result of DPAGCHE-enhanced image dataset. DPAGCHE-enhanced versions of the same images shown in Fig 5. The enhanced images demonstrate notable improvements in contrast, noise reduction, and clearer nucleus-cytoplasm boundaries, supporting better visual quality for subsequent classification.

Fig 5 and Fig 6 present a visual comparison between original cervical cell images and their DPAGCHE-enhanced counterparts. Fig 5 shows randomly selected images from the original dataset, while Fig 6 displays similar images after enhancement. Each category includes Normal, LSIL, and HSIL. By applying the DPAGCHE enhancement method, significant improvements in contrast, noise reduction, detail preservation, and nucleus-cytoplasm differentiation can be observed. Since the main focus of this study is automated classification, all results ultimately depend on how the algorithm interprets the images rather than human perception. Accordingly, image enhancement is conducted to explore whether the algorithm benefits from improved visual quality, particularly in terms of contrast, noise reduction, detail preservation, and nucleus-cytoplasm differentiation.

Contrast enhancement plays a critical role in improving image clarity. In the original images, particularly HSIL-024 and HSIL-085, the nucleus and cytoplasm have low contrast, making it difficult to distinguish their boundaries. In particular, DPAGCHE enhances contrast, resulting in darker and more distinguishable nuclei against the cytoplasm. Noise reduction is another observed improvement. The original dataset contains background noise, which may interfere with automated segmentation and classification. DPAGCHE reduces this noise, leading to a cleaner background and a more defined cellular structure. In LSIL-006, the application of a median filter successfully removes a linear shadow while preserving the nucleus’s structure and edges.

Another factor in image processing is detail preservation. Some enhancement techniques risk over-processing images, which can remove critical features. However, DPAGCHE, in combination with a median filter, retains nuclear textures and boundary definitions. This is particularly noticeable in LSIL and HSIL images, where the nuclear structure remains intact after enhancement. While these changes improve visual clarity, the impact on classification performance is the primary concern.

Since this study focuses on classification rather than image enhancement, no further analysis of the enhancement method is conducted. The goal is to determine whether visual improvements also translate into better classification performance. If the model achieves higher accuracy with enhanced images, it suggests that contrast adjustments and noise reduction improve feature extraction. In contrast, if there is no significant change, it indicates that visual clarity improvements may not directly benefit the deep learning model.

### Performance of the classification

Table 2 presents the key hyperparameters used in training the deep learning models for multiclass cervical cell classification. These hyperparameters were carefully selected to optimize the learning process and enhance the classification performance. The input image size was set to 224 × 224 × 3 to align with the standard input dimensions of pre-trained CNN architectures. Subsequently, a mini-batch size of 16 was chosen to balance memory usage and convergence speed. The training was conducted over a maximum of 25 epochs to prevent overfitting while allowing sufficient learning. The Adam optimizer, known for its adaptive learning capabilities, was utilized with an initial learning rate of 0.001. Meanwhile, the learning rate schedule was set to ‘piecewise,’ with a drop factor of 0.1 to gradually decrease the learning rate during training, enhancing fine-tuning. At the same time, Softmax activation was applied in the final classification layer to output class probabilities, while sparse categorical cross-entropy was employed as the loss function, suitable for multiclass classification tasks. Together, these hyperparameter settings aimed to improve the model’s generalization and robustness in distinguishing cervical cell categories.

**Table 2:** Hyperparameter settings used for the pre-trained models. Hyperparameters used to train all CNN models in this study, including image input size, batch size, optimizer, and learning rate schedule. These settings were selected to optimize convergence and reduce overfitting on the Herlev dataset for three-class cervical cell classification.

Hyperparameter	Parameter setting
Input size	224 × 224 × 3
Mini Batch size	16
Max Epoch	25
Activation function	Softmax
Optimizer	Adam
Initial Learning rate	0.001
Learn Rate Schedule	‘piecewise’
Learn Rate Drop Factor	0.1
Loss function	Sparse categorical cross-entropy

This study, similar to others in the field, confronts the ongoing challenge of evaluating classification models without universally agreed-upon performance benchmarks. Nevertheless, several clinical screening programs provide reference points for acceptable recall and specificity levels [14]. For instance, the UK Office for Health Improvement and Disparities recommends a minimum recall of over 90% for high-grade abnormalities, while Canada’s national screening guidelines suggest that at least 65% of positive Pap tests should be confirmed with precancerous or invasive lesions within 12 months. In our results, the best-performing model, ResNet50, achieved 85.38% recall and 91.63% specificity on the DPAGCHE-enhanced dataset. These figures suggest that the model approaches or meets the clinical expectations for high specificity and offers promising recall, especially considering the inherent class imbalance and the absence of post-classification rules or clinician feedback.

While these values may not yet fully reach the most stringent clinical thresholds for standalone deployment, they demonstrate significant potential as a decision support tool, particularly in resource-limited or high-throughput environments where manual screening is time-intensive. Furthermore, the notable improvements in F1-score and precision reinforce the model’s ability to balance recall with specificity, an important factor for minimizing false alarms and overdiagnosis. Future integration into clinical workflows could involve human-in-the-loop systems, where the model’s output serves as a triage mechanism or a second opinion to support cytotechnologists and pathologists.

This section focuses on identifying the CNN model that performs best for this classification task and evaluating the impact of DPAGCHE-enhanced images on various classification models compared to their performance using the original dataset. The models assessed include ResNet50, DenseNet201, InceptionV3, MobileNetV2, and Baseline CNN. Metrics such as accuracy, specificity, recall, precision, and F1-score were analyzed to determine the effectiveness of the DPAGCHE enhancement. Equations (7) to (11) are the according mathematical representation of each metric score.


$$Accuracy=\frac{\sum TP+\sum TN}{\sum TP+\sum TN+\sum FP+\sum FN}\times \text{100}$$ (7)

$$Sensitivity=\frac{\sum TP}{\sum TP+\sum FN}\times \text{100}$$ (8)

$$Precision=\frac{\sum TP}{\sum TP+\sum FP}\times \text{100}$$ (9)

$$Specificity=\frac{\sum TN}{\sum TN+\sum FP}\times \text{100}$$ (10)

$$F\text{1}-score=\frac{\text{2}\cdot \sum TP}{\text{2}\cdot \sum TP+\sum FP+\sum FN}\times \text{100,}$$ (11)

**Fig 7:**
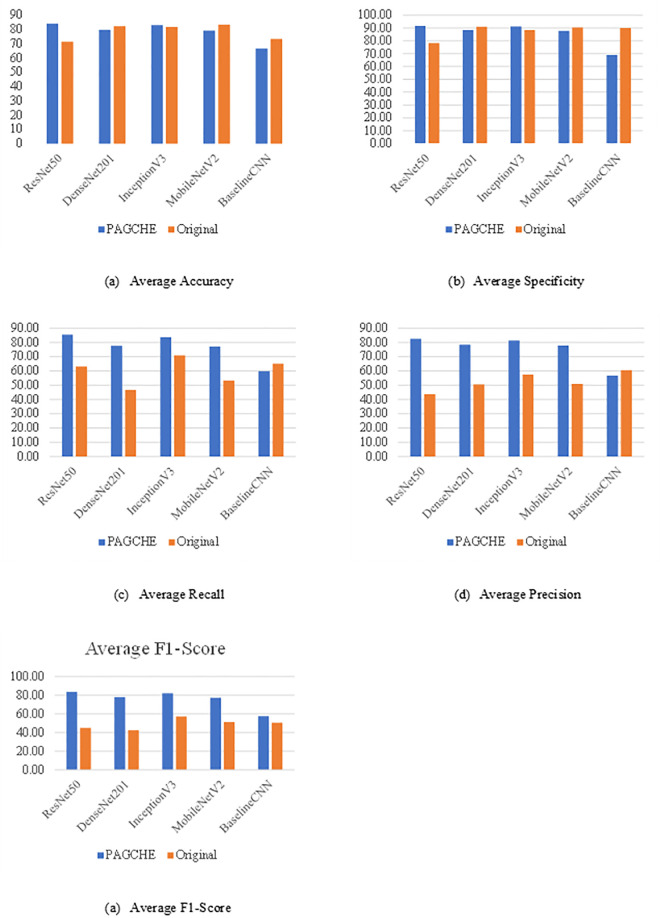
Bar Graph Comparison: CNN Model Metrics for DPAGCHE vs. Original Dataset. Bar graph comparison of classification metrics across five CNN models using the DPAGCHE-enhanced dataset (blue bars) versus the original dataset (orange bars). The enhanced images show significant improvements in recall, precision, and F1-score across all models, indicating better overall classification performance with DPAGCHE preprocessing.

Based on the result in Table 3, accuracy indicates the overall effectiveness of the classification models. The ResNet50 model demonstrates a substantial improvement of 12.70% with DPAGCHE, rising from 71.45% to 84.15%. This suggests that ResNet50 benefits significantly from the enhanced dataset. InceptionV3 also exhibits a slight improvement of 1.60%, highlighting some positive effects. However, DenseNet201 and MobileNetV2 display slight declines, with −3.05% and −5.10%, respectively, indicating that the enhancement may not be universally beneficial across all models. Baseline CNN presents a notable decrease of 9.29%, suggesting that simpler models may not capitalize on the enhancement as effectively. Specificity measures the true negative rate. This reflects how well the model identifies non-target instances. ResNet50 again shines with a 16.89% improvement, reaching 91.63% of the original 78.40%. InceptionV3 also benefits, with an increase of 3.04%. However, DenseNet201, MobileNetV2, and Baseline CNN exhibit declines, with Baseline CNN experiencing the most significant drop of 23.48%. These mixed results suggest that while DPAGCHE enhances specificity for some models, it might not be universally effective. Furthermore, recall measures the model’s ability to identify true positive instances. All models except Baseline CNN demonstrate improved recall with DPAGCHE, with DenseNet201 (65.82%), ResNet50 (35.14%), and MobileNetV2 (44.99%) demonstrating the most significant improvements. InceptionV3 also reveals a notable increase of 17.92%. Baseline CNN’s recall declines by 8.23%, further reinforcing the trend observed in the accuracy and specificity metrics. In addition, precision measures the accuracy of positive predictions. ResNet50 leads with an 89.52% improvement, followed by DenseNet201 (55.10%), MobileNetV2 (52.77%), and InceptionV3 (41.33%). Baseline CNN presents a slight decline of 6.24%. These results suggest that DPAGCHE significantly enhances the precision for most models, reflecting its effectiveness in improving the relevance of positive predictions. Moreover, the F1-score, a harmonic mean of precision and recall, provides a balanced measure of the models’ performance. ResNet50 (85.95%) and DenseNet201 (83.35%) indicate the most significant improvements. InceptionV3 (43.09%) and MobileNetV2 (51.94%) also demonstrate considerable gains. Baseline CNN demonstrates a modest improvement of 13.95%. Fig 7 display bar graph comparison based on the metrics score across different models. These results indicate that DPAGCHE enhances the overall performance of most models, particularly ResNet50 and DenseNet201, by balancing precision and recall. Generally, the analysis reveals that DPAGCHE-enhanced images generally improve the performance of advanced classification models.

**Table 3:** Performance metrics for the respective models. Classification performance of five CNN models on both the original and DPAGCHE-enhanced Herlev datasets. Metrics include accuracy, specificity, recall, precision, and F1-score. ResNet50 achieved the highest overall performance on the enhanced dataset, demonstrating significant improvements in all metrics compared to its performance on the original dataset.

Model	Dataset	Accuracy	Specificity	Recall	Precision	F1-Score
ResNet50	DPAGCHE	84.15	91.63	85.38	82.57	83.51
	Original	71.45	78.40	63.17	43.58	44.91
DenseNet201	DPAGCHE	79.78	88.49	77.61	78.34	77.83
	Original	82.29	91.07	46.81	50.51	42.45
InceptionV3	DPAGCHE	83.06	91.19	83.65	81.14	81.97
	Original	81.75	88.50	70.93	57.42	57.29
MobileNetV2	DPAGCHE	79.20	87.83	77.03	77.87	77.40
	Original	83.45	90.49	53.13	50.97	50.95
Baseline CNN	DPAGCHE	66.67	68.99	59.80	56.72	57.55
	Original	73.50	90.17	65.17	60.50	50.50

ResNet50 benefits the most across all metrics, demonstrating significant improvements in accuracy, specificity, recall, precision, and F1-score. DenseNet201 also suggests notable improvements in recall, precision, and F1-score, albeit with slight declines in accuracy and specificity. In contrast, InceptionV3 and MobileNetV2 provide mixed results, with improvements in some metrics and declines in others. Baseline CNN consistently presents performance declines, suggesting that simpler models may not fully leverage the benefits of DPAGCHE enhancements. Overall, the DPAGCHE-enhanced dataset appears to significantly improve the performance of complex models like ResNet50 and DenseNet201. However, its effectiveness varies across different models, indicating that the enhancement’s benefits may depend on the model’s architecture and complexity. Therefore, future research could focus on optimizing DPAGCHE for various model types to maximize its performance-enhancing potential across the board.

To evaluate the statistical significance of image enhancement using the DPAGCHE method, we conducted a paired t-test comparing the classification results of ResNet50 on the original and enhanced images. The model was executed ten times for each image type, and the mean and standard deviation of five evaluation metrics were calculated. Table 4 summarizes the results, including the mean differences and corresponding p-values.

**Table 4:** Paired t-test comparison between ResNet50 performance on original images and DPAGCHE-enhanced images (n = 10 repetitions). The results show that DPAGCHE-enhanced images significantly improved model performance across all metrics, with all p-values < 0.001.

Metric	DPAGCHE (Mean ± Std)	Original (Mean ± Std)	Mean Difference	p-value
Accuracy	85.31 ± 4.07	60.20 ± 11.10	25.11	0.0001
Specificity	90.80 ± 3.43	61.70 ± 15.71	29.10	0.0004
Recall	82.60 ± 1.07	59.30 ± 15.54	23.30	0.0007
Precision	81.90 ± 1.29	47.50 ± 5.91	34.40	< 0.0001
F1-Score	85.30 ± 2.54	48.80 ± 6.66	36.50	< 0.0001

As presented in Table 4, the ResNet50 model trained on DPAGCHE-enhanced images consistently outperformed the original images across all evaluation metrics. The Accuracy improved significantly from 60.20 ± 11.10 to 85.31 ± 4.07, showing a mean increase of 25.11 points (p = 0.0001), indicating better overall classification performance. Specificity increased by 29.10 points (p = 0.0004), suggesting the enhanced images helped the model better identify negative cases. Similarly, Recall improved from 59.30 ± 15.54 to 82.60 ± 1.07 (p = 0.0007), reflecting enhanced sensitivity in detecting positive cases. The most notable gains were seen in Precision and F1-Score, which rose by 34.40 and 36.50 points respectively, both with p-values < 0.0001, indicating a much more balanced and reliable classification. Overall, the consistently low p-values (< 0.001) across all metrics confirm that the observed performance improvements with DPAGCHE enhancement are statistically significant and not due to random chance.

The results demonstrate that the DPAGCHE enhancement significantly improves classification performance across all evaluation metrics. The largest improvement was observed in the F1-Score, with a mean increase of 36.5 points, followed by Precision and Specificity. All p-values are well below the 0.05 threshold, indicating statistically significant differences. These findings confirm that the enhanced images produced by DPAGCHE contribute positively to the performance of the ResNet50 model. Hence, ResNet50 was selected for in-depth validation due to its superior and consistent performance compared to other evaluated models.

To determine the effectiveness of the DPAGCHE-enhanced dataset compared to the original dataset, we calculated the overall percentage improvement for each performance metric (Accuracy, Specificity, Recall, Precision, and F1-score) across all models and the result of percentage improvement displayed in Table 5. The formula used for calculating the percentage improvement is:

**Table 5:** Improvement of model performance with DPAGCHE enhancement. Percentage improvement of each classification metric when using DPAGCHE-enhanced images compared to the original dataset. The most substantial gains were observed in F1-score (+53.65%) and precision (+44.29%), indicating improved model balance and reduced false positives. A modest gain in accuracy (+7.86%) and recall (+28.17%) further supports the effectiveness of the enhancement, although specificity slightly declined (−1.94%), suggesting a trade-off between sensitivity and true negative detection.

Metrics	Accuracy	Specificity	Recall	Precision	F1 Score
Percentage Improvement	7.86%	−1.94%	28.17%	44.29%	53.65%


$$PercentageImprovement=\left(\frac{Average(PAGCHE)-Average(Original)}{Average(Original)}\right)$$ (12)

**Fig 8:**
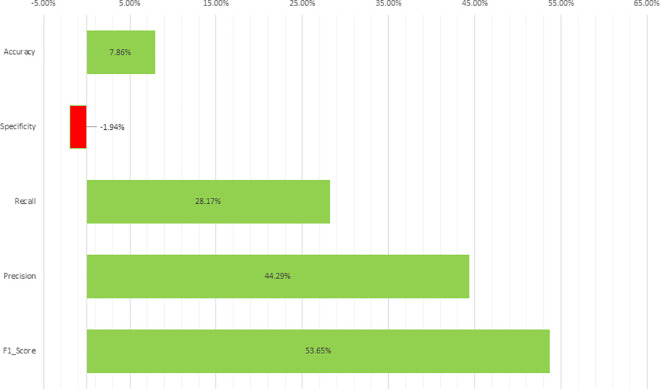
Visual representation of comparative improvements in model performance with DPAGCHE enhancement. Illustrating Relative Percentage Changes; Green Bars Indicate Performance Gains, While Red Bars Represent Performance Declines.

Fig 8 illustrates the percentage improvement achieved by the DPAGCHE-enhanced dataset compared to the original dataset across various performance metrics, including F1-score, precision, recall, specificity, and accuracy. This comparative analysis aims to assess the impact of the DPAGCHE enhancement method on the overall classification performance for cervical cell images. In particular, the green bars represent the percentage increase in metrics, while the red bar signifies a decrease, providing a clear visual representation of performance gains and potential trade-offs.

The results demonstrate a notable improvement in F1-score, precision, and recall, with the highest percentage gain observed in F1-score at 53.65%. This substantial increase highlights the effectiveness of the DPAGCHE enhancement in balancing precision and recall, which is crucial for multiclass classification tasks where accurate differentiation between classes is challenging. Precision also improved by 44.29%, indicating a reduction in false positives, while recall increased by 28.17%, reflecting enhanced recall in detecting cervical cell abnormalities.

Interestingly, the figure shows a slight decline in specificity, with a negative improvement of −1.94%. This drop suggests that the enhanced dataset may have introduced some challenges in correctly identifying true negatives, possibly due to increased recall leading to a minor trade-off in specificity. However, the overall accuracy improved by 7.86%, demonstrating the DPAGCHE-enhanced dataset’s ability to enhance the general classification performance. These findings underscore the significance of preprocessing in deep learning-based medical image classification and suggest that DPAGCHE enhancement can significantly contribute to more accurate and balanced classification outcomes.

### Performance based on cell class

**Fig 9:**
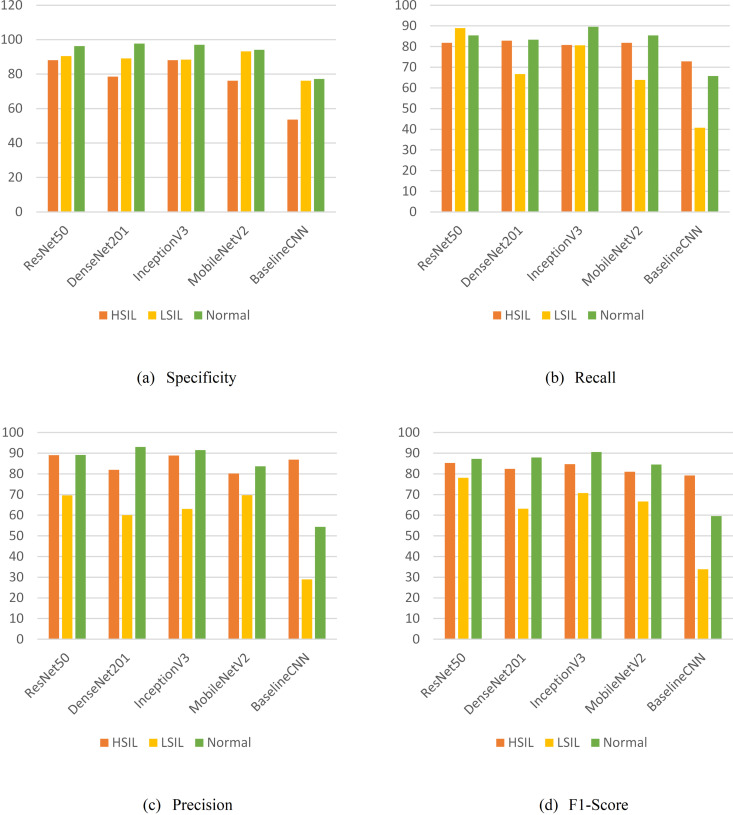
Metrics values for each model across HSIL, LSIL, and normal classes.

Fig 9 illustrates a class-wise comparison of specificity, recall, precision, and F1-score for three cervical cell classification categories: HSIL, LSIL, and Normal cells. The comparison is conducted across five CNN models: ResNet50, DenseNet201, InceptionV3, MobileNetV2, and a Baseline CNN as below:

- Specificity (Fig 9a): ResNet50 and InceptionV3 demonstrated high specificity across all classes, with InceptionV3 slightly behind ResNet50 in LSIL yet comparable overall. DenseNet201 had the highest specificity for the Normal class (97.78%) yet lower for HSIL (78.57%). MobileNetV2 performed well in LSIL (93.2%) and Normal (94.1%) yet lower in HSIL (76.2%). Baseline CNN had the lowest specificity, particularly notable in HSIL (53.57%) and LSIL (76.19%), indicating significant room for improvement.
- Recall (Fig 9b): ResNet50 demonstrated strong recall in all classes, particularly in LSIL (88.89%). DenseNet201 exhibited better recall in HSIL (82.83%) but struggled with LSIL (66.67%). InceptionV3 performed well, especially in Normal (89.58%). MobileNetV2 had a moderate recall, with a notable drop in LSIL (63.9%). Baseline CNN presented the lowest recall across all classes, particularly in LSIL (40.74%).
- Precision (Fig 9c): ResNet50 excelled in precision, particularly in HSIL (89.01%) and Normal (89.13%). InceptionV3 also resulted in high precision, though slightly lower in LSIL (63.04%). DenseNet201 and MobileNetV2 had moderate precision, with DenseNet201 performing well in Normal (93.02%). Baseline CNN displayed significant variability, performing well in HSIL (86.87%) yet poorly in LSIL (28.95%).
- F1-Score (Fig 9d): ResNet50 achieved the highest F1-scores across all classes, indicating a good balance of precision and recall. InceptionV3 also performed well, especially in Normal (90.53%). DenseNet201 demonstrated a balanced performance, particularly in HSIL (82.41%) and Normal (87.91%). MobileNetV2 had moderate F1-scores, with the lowest performance in LSIL (66.7%). Baseline CNN presented the lowest F1-scores, particularly for LSIL (33.85%).

In summary, ResNet50 emerged as the top-performing model with strong metrics across specificity, recall, precision, and F1-score, particularly excelling in HSIL and Normal classes. Meanwhile, InceptionV3 and MobileNetV2 demonstrated reliable performance with areas for enhancement. DenseNet201, despite its high specificity, struggled with recall, particularly for LSIL, suggesting a need for a better balance between recall and specificity. At the same time, Baseline CNN highlighted the advancements made by more sophisticated models, serving as a benchmark for improvement. Collectively, this critical analysis underscores the significance of selecting models based on the specific needs of the classification task, with a particular emphasis on balancing precision, recall, and overall effectiveness for accurate cervical cell classification.

## Conclusion

This study evaluated the performance of five deep learning models—ResNet50, DenseNet201, InceptionV3, MobileNetV2, and a Baseline CNN—for multiclass classification of cervical cell images. These models were assessed on both the original and DPAGCHE-enhanced datasets to investigate the impact of preprocessing on classification performance. The results demonstrated that the DPAGCHE-enhanced dataset significantly improved classification metrics across all models, particularly in recall, precision, and F1-score. Compared to the original dataset, the enhanced images led to a + 7.86% increase in accuracy, + 28.17% in recall, + 44.29% in precision, and +53.65% in F1-score, though specificity experienced a –1.94% decrease. Notably, ResNet50 achieved the best performance with 84.15% accuracy, followed closely by InceptionV3 at 83.06%. ResNet50’s superior results are attributed to its residual connections, which mitigate the vanishing gradient issue and enable deeper feature extraction, allowing it to effectively handle the complex patterns and morphological variations in cervical cell images. In contrast, the Baseline CNN achieved only 66.67% accuracy, reflecting the limitations of shallow architectures in medical image classification tasks.

Despite these enhancements, the study highlighted several challenges. The issue of class imbalance significantly influenced the classification performance, particularly in the recall and F1-scores of minority classes such as LSIL. The uneven distribution of the Herlev dataset caused models to be biased toward majority classes, reducing their ability to accurately detect underrepresented categories. Although data augmentation techniques such as flipping, shifting, and rotation were applied to enhance dataset variability, they did not fully mitigate this imbalance. Future studies could address this limitation by incorporating resampling strategies (e.g., oversampling minority classes or undersampling majority classes), or implementing class weighting in the loss function to penalize misclassifications of underrepresented classes more heavily. In terms of computational complexity, models like ResNet50 and DenseNet201, while yielding superior classification metrics, required substantial training time and memory resources. These requirements may limit the practicality of deploying such models in resource-constrained clinical settings. To reduce computational demands, future work could explore model pruning, quantization, or the use of efficient architectures such as EfficientNet or MobileNetV3, which maintain competitive performance while significantly reducing inference cost.

Another key limitation of this study is the exclusive use of the Herlev dataset, which may impact the generalizability of the findings. While the Herlev dataset is widely used and well-annotated, it represents a controlled and relatively small sample with limited variability in staining protocols, imaging conditions, and demographic diversity. This introduces a risk of overfitting, especially when using deep models like ResNet50 and DenseNet201, which are highly expressive and prone to memorizing dataset-specific features. To enhance the robustness of future models, external validation using independent cervical cell image datasets (e.g., SIPaKMeD or CRIC Cervix) is recommended. Such validation would assess the models’ adaptability to images acquired from different sources or with different imaging parameters. Additionally, techniques like cross-dataset evaluation, domain adaptation, and transfer learning with fine-tuning could be explored to improve generalizability. We acknowledge this limitation and consider it a crucial area for future extension of this work.

In conclusion, this research demonstrates the potential of deep learning models, particularly ResNet50 with DPAGCHE-enhanced preprocessing, for improving cervical cell classification accuracy and reliability. In particular, ResNet50’s robustness in extracting deep hierarchical features, combined with the noise-reducing and contrast-enhancing effects of DPAGCHE preprocessing, makes it the optimal model in this study. However, challenges such as class imbalance, recall to image quality, and computational efficiency remain critical areas for further exploration. Therefore, future work could investigate advanced techniques, such as synthetic data generation, hybrid architectures, and transfer learning, to improve generalization, enhance minority class recall, and optimize computational efficiency. Nonetheless, these improvements could contribute to developing more accurate and scalable automated cervical cancer screening systems, ultimately aiding in early detection and reducing global cervical cancer mortality.
